# A process evaluation of ‘We Can Quit’: a community-based smoking cessation intervention targeting women from areas of socio-disadvantage in Ireland

**DOI:** 10.1186/s12889-022-13957-5

**Published:** 2022-08-10

**Authors:** Catherine D. Darker, Emma Burke, Stefania Castello, Karin O’Sullivan, Nicola O’Connell, Joanne Vance, Caitriona Reynolds, Aine Buggy, Nadine Dougall, Kirsty Loudon, Pauline Williams, Fiona Dobbie, Linda Bauld, Catherine B. Hayes

**Affiliations:** 1 Public Health & Primary Care, Institute of Population Health, School of Medicine, Trinity College Dublin, Dublin, Ireland; 2 Irish Cancer Society, Dublin, Ireland; 3 Health Promotion and Improvement, Health Service Executive, Dublin, Ireland; 4 School of Health & Social Care, Edinburgh Napier University, Edinburgh, Scotland; 5 Freelance Researcher, London, UK; 6 Patient/Public Representative, London, UK; 7 College of Medicine, Usher Institute and SPECTRUM Consortium, University of Edinburgh, Edinburgh, Scotland

**Keywords:** Smoking cessation, Behavioural intervention, NRT, Deprivation, Women, Trials, Qualitative, Process evaluation

## Abstract

**Background:**

Smoking poses a serious risk of early preventable death and disease especially for women living with socio-economic disadvantage (SED). A smoking cessation programme, ‘We Can Quit’, was developed in Ireland tailored to SED women. This includes group-based support delivered by trained lay local community facilitators (CFs) and free nicotine replacement therapy (NRT). The intervention was pilot tested in a cluster randomised controlled trial, ‘We Can Quit 2’. This paper reports on the WCQ2 process evaluation which assessed feasibility and acceptability of the programme and trial processes.

**Methods:**

Embedded qualitative design using the UK Medical Research Council’s process evaluation framework. Semi-structured interviews with trial participants (*N* = 21) and CFs (*N* = 8). Thematic analysis was utilised.

**Results:**

Peer-modelling, a non-judgemental environment, CFs facilitation of group support were viewed as acceptable programme related factors. Some participants expressed concerns about NRT side effects. Provision of free NRT was welcomed and accepted by participants, although structural barriers made access challenging. Pharmacists took on a role that became larger than originally envisaged – and the majority provided additional support to women in their quit attempts between group meetings which augmented and supplemented the intervention sessions provided by the CFs. Participants reported good acceptance of repeated measures for data collection, but mixed acceptability of provision of saliva samples. Low literacy affected the feasibility of some women to fully engage with programme and trial-related materials. This was despite efforts made by intervention developers and the trial team to make materials (e.g., participant intervention booklet; consent forms and participant information leaflets) accessible while also meeting requirements under 2018 European General Data Protection Regulation legislation. Hypothetical scenarios of direct (e.g., researcher present during programme delivery) and indirect (e.g., audio recordings of programme sessions) observational fidelity assessments for a future definitive trial (DT) were acceptable.

**Conclusions:**

Intervention and trial-related processes were generally feasible and acceptable to participants and CFs. Any future DT will need to take further steps to mitigate structural barriers to accessing free NRT; and the established problem of low literacy and low educational attainment in SED areas, while continuing to comply within the contemporary legislative research environment.

**Trial registration:**

WCQ2 pilot trial (ISRCTN74721694).

**Supplementary Information:**

The online version contains supplementary material available at 10.1186/s12889-022-13957-5.

## Background

Tobacco use is the main cause of preventable death worldwide [1] and has been causally related to a variety of chronic diseases and fourteen types of cancer [2], including lung cancer [3]. In Ireland, as in most high-income countries, smoking prevalence and associated health consequences are greater in socioeconomically disadvantaged (SED) populations [4–6]. Social determinants that exacerbate health inequalities are associated with psychosocial factors, such as high daily stress, lack of social support, and pro-smoking social norms [7–9].

Gender is also a determinant of smoking [10]. A review of evidence from effectiveness trials have indicated that women are less likely to quit smoking and have greater difficulty maintaining long-term smoking abstinence than men [11]. In Ireland, this is reflected in increased lung cancer incidence among women between 1994–2015. Lung cancer is now the main cause of mortality from cancer in women in Ireland [12, 13].

Smoking in women is related to SED [14]. The link between disadvantage, gender and smoking status is recognised by the World Health Organization (WHO) Framework Convention on Tobacco Control that argues tobacco control strategies should be tailored to disadvantaged women to reduce smoking prevalence and associated illness [4]. These strategies should address individual aspects of smoking and socio-economic factors [10, 15].

Social support has been recognised as facilitating smoking cessation [16]. Smokers from SED groups, and women in particular, usually experience a lack of social support for smoking cessation from their personal environment and from available cessation aids [7, 9, 10]. Addressing social support needs of SED women may be key for improving smoking cessation [10, 17].

Group-based behavioural interventions involve the delivery of behavioural techniques, specific advice, and support from other participants [18]. Although group support is more effective than self-help, more evidence is needed to determine its effectiveness compared to intensive individual counselling and in sub-groups of smokers [19], such as SED women. To date, the evidence on the effectiveness of group-based smoking cessation interventions tailored to women is scarce [20–22]. Only one previous randomised controlled trial (RCT) has evaluated a group-based cessation intervention tailored to the specific needs of disadvantaged African-American women, with positive abstinence rates [20]. Findings from other studies have shown that the use of nicotine replacement therapy (NRT) increases the rate of quitting by 50% to 60%, regardless of setting [23], and can help to prevent smoking relapse [24]. However, the cost of NRT has hindered access and potential benefits to SED smokers [9, 25].

We Can Quit2 (WCQ2) study was a pilot cluster RCT conducted in four matched pairs of SED districts in Ireland. It set out to evaluate the feasibility and acceptability of We Can Quit (WCQ), a community-based intervention to address smoking cessation in women delivered by trained lay community facilitators (CFs) [26, 27]. It was based on the Socio-Ecological Model (SEM) [28] and developed using a community-based participatory research approach [29]. The detailed trial methodology and primary quantitative results of the WCQ2 pilot study are described elsewhere [30].

Trial evaluations typically focus on understanding whether interventions are effective but cannot explain how and why interventions succeed or fail in attaining outcomes. This is particularly important to definitive trials (DTs) of complex interventions [31]. Of growing importance is the need to understand why interventions succeed or fail in the pilot trial phase (such as WCQ2), thereby allowing earlier design adaptations before progression to DT [32]. A process evaluation, as outlined by the UK Medical Research Council (MRC) [31], provides a framework for assessing an intervention’s implementation, the identification of contextual factors and proposed mechanisms for change. It is considered an essential part of designing and testing complex interventions and complements earlier UK MRC guidance [33]. Hence, a qualitative, mixed-method process evaluation was embedded into the WCQ2 trial, following UK MRC specific guidance [31]. To our knowledge few smoking cessation feasibility trials have applied UK MRC process evaluation guidance, with only one completing a process similar to the current study [34]. Others examined acceptability of the cessation intervention only from the perspectives of participants, overlooking the assessment of trial processes acceptability [35, 36].

In this paper, we expand upon this important area and take an in-depth approach investigating programme factors (group based delivery, role of community facilitators, free NRT) while also taking into account how the intervention interacted with the context of the participants (women from SED with low literacy) and the context in which the trial was implemented (General Data Protection Regulations (GDPR) 2018 [37] legislation relating to trial documentation).

## Methods

### Design

This research is embedded within a larger trial which took the philosophical stance of ‘pragmatism’, which is the most commonly stated philosophy supporting mixed methods research [38–41]. Pragmaticism values both objective and subjective knowledge, and investigators using both quantitative and qualitative data, adopt a postmodern viewpoint and employ a reflective lens of the social, environmental, and other contexts at play. In this tradition, knowledge is constructed using data through the adoption of an inductive-deductive logic, thereby increasing the credibility of the research findings [39]. This aspect of the trial embraces a qualitative research design, using face-to-face individual and paired interviews. An inductive approach, where the research team attempted to make sense of context and data without imposing pre-existing expectations on the topic under inquiry, was used [42]. Stakeholder interviews are a common method of inquiry as outlined by the UK MRC’s framework to ‘capture emerging changes in implementation, experiences of the intervention and unanticipated or complex causal pathways’ [31]. The School of Medicine Research Ethics Committee, Trinity College Dublin, approved this study (Reference number 20170404). All research procedures have been performed in accordance with the Declaration of Helsinki.

### WCQ2 pilot trial overview

Participants were recruited in four consecutive waves, each one in a matched SED district [27]. Treatments were the WCQ intervention, which comprised 12 weeks of group-based behavioural support and optional access to combination NRT [43] without charge for all women (e.g., patches, with either inhalator, gum, lozenges or spray). The WCQ intervention also included advice from community pharmacists to support NRT use (e.g., titration of NRT amounts). In Ireland, patients entitled to the General Medical Scheme (GMS) are eligible for low or no cost prescriptions[44], while non-GMS ‘private’ patients typically pay directly for NRT. CF activities focused on increasing self-efficacy; on peer-support by sharing experiences at sessions and celebrating achievements with family, friends, and the local community [26, 27]. WCQ participants also received an intervention booklet which included fact sheets, activity worksheets, a handheld NRT record, and signposting information. They were invited to keep a smoking journal to use as a personal space for reflections from the first session to increase their understanding of their smoking behaviour.

### Selection of participants

A purposive sampling procedure was employed, targeting key stakeholders involved in the trial. The focus of recruitment was to identify and select information-rich cases [45] from whom it was possible to learn about experiences of programme recipients, the facilitators who delivered the intervention and to elucidate participants’ experiences of being involved in a pilot RCT. Key participant characteristics and outcome assessment at follow up, including self-reported smoking behaviours at baseline are shown in Table 1.

**Table 1:** Baseline socio-demographic and smoking characteristics of We Can Quit intervention participants who were interviewed and outcome assessment at 12-week follow-up interview (*N* = 21)

Socio-demographics	
***Age*** mean, (SD)	52.1, (10.7)
***Marital Status***	*n (%)*
Married or cohabiting	11 (52.4)
Not married (single, separated, divorced, widowed)	10 (47.6)
***Education***	
No formal / Primary / Lower	8 (38.1)
Secondary / Technical or Vocational / Completed Apprenticeship	8 (38.1)
Degree (Diploma, Masters, PhD)	5 (23.8)
***Employment***	
Full/part time	8 (38)
Not in paid employment	13 (62)
***General Medical Scheme (GMS) entitled patients or General Practitioner card***^**c**^	
Yes	15 (71.4)
No	6 (28.6)
**Smoking behaviour at baseline**	
***Reasons for smoking***	
For pleasure / to cope	6 (28.6)
Habit / Addicted / Other	15 (71.4)
***Time after waking before first cigarette***	
Within 5 min	14 (66.6)
After 5 min	7 (33.3)
***Determination to give up smoking***	
Not at all determined	0
Quite determined	6 (28.6)
Very / Extremely determined	15 (71.4)
**We Can Quit intervention delivery**	
***Attendance at sessions***	
Between 1 and 8 sessions	8 (38)
Between 9 and 12 sessions	13 (62)
***Used Nicotine Replacement Therapy during intervention delivery***^**a**^	
Yes	12 (57.1)
No	6 (28.6)
***Smoking status at 12-weeks (end of programme)***^**b**^	
Abstinence	8 (38)
Continued smoking	13 (62)

^a^ Three participants did not give any information on NRT use

^b^ Corroborated by saliva tests

^c^ General Medical Scheme (GMS) entitled patients are eligible to access primary care services free of charge and are eligible for low or no cost prescriptions. Those patients with a General Practitioner (GP) card are eligible to see their GP free of charge

### Description of community facilitators (CFs)

The CFs selected by the WCQ delivery partners, belonged to or worked in the community where they delivered the training. Most (seven out of eight) were ex-smokers. Three were full time professionals across areas such as family support, local development programmes (e.g., a community worker role) and/or adult education. Their time spent working on the WCQ programme was covered by their employer.

All CFs were trained to the National Standard in Smoking Cessation [46] and CFs in Wave 4 were also trained in group facilitation skills (comprising two days of training). Facilitators in Wave 1 had previous experience in delivering the original WCQ pilot programme in a different community setting. For Waves 2, 3 and 4, it was their first time delivering the programme. All CFs were women.

### Procedure

At the end of the programme, all participants who attended at least one group session were contacted by telephone and invited for interview. A semi-structured interview schedule allowed for probing, follow-up questions and flexibility. Interview schedules were piloted. (See Additional Files 1 and 2 for sample interview schedules for participants and CFs). Interviews were face-to-face and occurred between June 2018 and May 2019 at times and locations convenient to participants. Only the interviewer (EB; female; MSc-level training; full-time trial research assistant) and interviewees were present. The interviewer was known to interviewees at the time of interviews from previous contact regarding recruitment and follow up within the trial. Each interview lasted on average 20–30 min, while CF interviews lasted approximately an hour. Participant interviews were conducted individually, while interviews with CFs (two CFs per intervention site) were conducted together. Interviews were audio recorded and transcribed verbatim by a professional transcriber. Observational field notes were completed to enhance data and provide context for analysis. A participant information leaflet (PIL) was provided to participants. Informed written consent was obtained prior to commencing interviews and participation was voluntary. Efforts were made to explain complex terminology in layperson’s language in the consent form and the PIL by also engaging with the National Adult Literacy Agency (NALA) [47]. A necessary balance was needed in order to include sufficient detail to comply with legislation such as GDPR [37]. The Research Assistant (EB) verbally explained all trial processes to participants to maximise informed consent. The PIL and consent forms were given to each participant at least 24 h before signing, affording participants time to review.

To ensure anonymity, participants were given identification tags (e.g., W1-CF1, which corresponds to Wave 1 of recruitment, Community Facilitator 1; W3-P0004, which corresponds to Wave 3 of recruitment, participant number 0004). Reporting of the study methods have followed published standards for undertaking and reporting qualitative research (COREQ) [48].

### Data analysis

Thematic analysis, a recognised method to identify, analyse, organise, describe, and report themes found within qualitative data, was used [49]. Data were coded in six phases: familiarisation with data, generating initial codes, searching for themes among codes, reviewing themes, defining and naming themes through the production of a ‘coding frame’, and producing the final analyses through the application of the coding frame to available data [49]. The use of a coding frame allows for the organisation of codes, to encourage trustworthiness of the data through each phase of thematic analyses [50]. NVivo version 12 software was used to organise data into themes and nodes.

Three researchers (CD, KOS & EB) independently read all transcripts. Rigorous line-by–line coding was applied, with a focus on experiential claims and concerns. Data patterns were clustered into a thematic structure to identify and categorise major themes and sub-themes. Data saturation was achieved when no new codes or themes emerged within the analyses [51]. Any differences in interpretation were resolved through discussion. A fourth independent researcher (JI) with qualitative expertise, reviewed the coding frame and applied it to approximately 10% of transcripts, improving analytical triangulation [52]. Transcripts were not returned to participants.

## Results

Of 50 women invited, 21 were interviewed (this corresponded to a total of 3, 7, 5 and 6 women from Waves 1 to 4 respectively; 41% response rate) within the timeframe (one to two weeks post final programme session). The full cohort of CFs were interviewed, two in each of the four intervention sites, resulting in a total of eight CFs interviews.

**Fig. 1:**
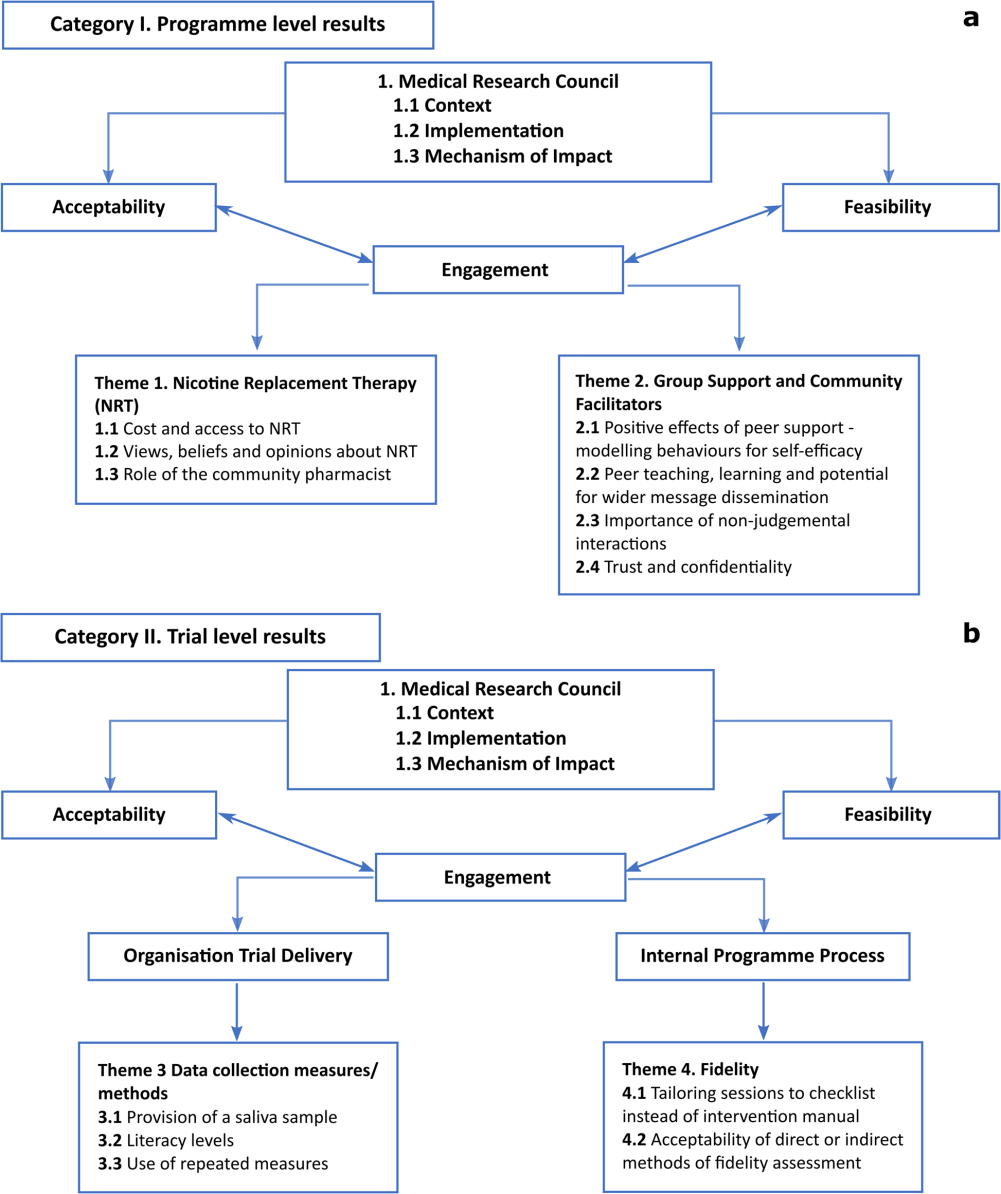
Coding frame for the qualitative results, categorised into **(a)** ‘Programme level’ and **(b)** ‘Trial level’ results following the UK MRC process evaluation framework

Figure 1 displays the overall coding frame for the qualitative results, categorised into a) ‘Programme level’ and b) ‘Trial level’ results following the UK MRC process evaluation framework [31].

### Category I. Programme level results

Two main themes were identified under this category: NRT and group support.

### Theme 1. Nicotine replacement therapy (NRT)

#### Subtheme 1.1. Cost of and access to NRT

In the WCQ2 trial, the cost of NRT for non-GMS patients was covered by the Irish Cancer Society. This was seen as acceptable and appreciated by participants.

W4–P049: *It was great [free NRT], yeah, yeah, I found it fantastic. It was great to get it.*

However, GMS-entitled participants were required to obtain an NRT prescription before it could be dispensed without charge. In some circumstances, this created a feasibility problem because of a lack of available general practitioner (GP) appointments and could also result in the participant feeling uncomfortable when engaging with the dispensing pharmacy.

W4–CF 2: *… one of the ladies said sure ‘I can’t even get an appointment; it takes 3 weeks to get an appointment’…*

W4–CF 1: *And then when the pharmacists confronted the ladies about the prescription they kind of were uncomfortable that they felt em they were being put under a bit of pressure to get the prescription off their doctor and they were stressing over it.*

#### Subtheme 1.2. Views, beliefs, and opinions about NRT

Some participants expressed concerns about using NRT. Some concerns were associated with views that NRT can make the user feel ill.

W3–P0005: *I never felt sick from cigarettes. It’s (the patch) making me sick and sometimes I’m afraid that when I’m putting the patch on I’m scared that this is going to make me sick.*

Other concerns related to its perceived potential for dependence.

W4–P065: *Yeah, and I’m still having to use the nicotine replacement there now and I’m still dependent on that. I’d had a big worry about getting addicted to this (inhaler)…I reach for it, just like I used to reach for a cig.*

#### Subtheme 1.3. Role of the community pharmacist

A key aspect of the WCQ2 trial was to bring clarification on NRT and its role in smoking cessation. To this end, efforts were made in preparatory phases to identify one local community pharmacy in each of the four study areas willing to dispense and provide information and support to the women on their quit attempts.

W1–P0007*: You see the pharmacist coming in like giving an account of what everything does and how you come off it and how you cut down and all like that would be a big help. Yeah, he was very good, his attitude was really good, and he couldn’t have been more helpful like do you know.*

However, some pharmacists were going beyond traditional roles of dispensary pharmacy and were providing participants with additional brief interventions that may have augmented group sessions when they presented at the pharmacy for their NRT. It also became apparent that some CFs actively encouraged participants to link with pharmacists if they were struggling with their quit attempt or lulls in motivation between group meetings. This was seen as acceptable by participants.

W2–CF 1: ….*they had their moments and they’d arrive in the door to him…And he’d [pharmacist] a little room to the side and he’d take them in and talk it through with them. The chat with the pharmacist really kept them going in their quit attempts. They’d arrive down to him sometimes in a panic.*

However, not all pharmacists were as supportive. For example, an optional component of the programme included CFs inviting pharmacists to attend a group session to explain NRT, however, not all were available or willing to do this.

W4–CF 2: *No, the pharmacist didn’t come in because they couldn’t, they didn’t want to stand up and talk in front of people.*

### Theme 2. Group support and community facilitators

#### Subtheme 2.1. Positive effects of peer support – modelling behaviours for self-efficacy

Participants were very accepting of role-modelling behaviours which demonstrated that stopping smoking was possible which featured as part of the group sessions.

W3–P0005: *Going to the meetings…you’re more aware of where you were smoking, who was around you…and then by listening to the other people, how they did it, you pick up all the little knick knacks like you know.*

The ability to relate and to recognise oneself within a group is a core tenet of why group support works. Trust and compatibility underpin this and the related concept of learning from others.

W2–P0041: *Well, I found when I came first that everybody was the same as me…You only just felt we’re all here together on the same wavelength…. Normally when I give up the cigarettes, I feel that somebody has after gone from my life, I’m after losing a friend, I’d be pining but this time I says, ‘no I’m not losing a friend’. So, something worked in the head.*

Participants’ spoke of embracing and accepting group support in terms of building capacity by increasing their skills, self-efficacy, and support for maintaining abstinence. The group support they received strengthened and reinforced their intentions to cease or decrease smoking.

#### Subtheme 2.2. Peer teaching, learning and potential for wider message dissemination

In practice, participants often provided informational support to one another, offering advice and suggestions about smoking cessation strategies through an informal exchange process.

W1-P0040: …*that lady she taught me one thing that I didn’t know, and I taught her something that she wouldn’t have known… we all found out something different to help us and if one fell off the wagon we’d turn around and say, ‘don’t worry about it’.*

Participants reflected that their relationships with members of the group became a part of their motivation to quit:

W3-P0003: *I feel like if I went back smoking I’d be letting them down… it’s not about letting myself down, it’s about letting them down.*

Through shared experience, participants demonstrated empathy, which went deeper than the standard ‘common bond in common disease’, as outlined here:

W3–CF 1: *…it became a nice comfortable space to be in and I think that’s what encouraged them to come back. Yes, and for the weeks where they were feeling a bit vulnerable and a bit low and a bit judge[d] and self-berating, the other women in the group expressed their encouragement and compassion.*

#### Subtheme 2.3. Importance of non-judgemental interactions

Participants described the support group environment as being an accepting non-judgmental one where they felt understood. This was in contrast to attitudes some had encountered from loved ones.

W2-P0026: *…because I think they understood what you were going through…people at home were great and they were supportive but they [‘re] thinking after a day or two ‘you should be over it’, whereas this they knew what you were going through. So, we kind of all went through it together.*

Most participants expressed that group sessions enhanced the feasibility of them persisting with their quit attempt:

W1-P0004: *… it’s a long-term thing, …it’s still one day at a time ok but I feel like there’s a spell broken, that’s the only way I can explain it, that smoking, or addiction is a spell, it’s like being in a spell and that’s broken, which is huge.*

#### Subtheme 2.4. Trust and confidentiality

A sense of trust was built up to such an acceptable level that participants reported feeling psychologically safe enough to be vulnerable and honest.

W4–P010: *We were quite an open group. The kind of type of women just wearing our life on our sleeve and just say what we had to say.*

Women reported freedom to discuss their general life stresses and the stress experienced *vis-a-vis* making a quit attempt.

W2-P0011: *Yeah I didn’t hide it because it was so private. I wasn’t going to lie and say everything was great because we all had a good rant every now and again.* ….*somebody was going through the same, they were really close to tears, and just to see that and go, “right I’m not cracking up, I’m not losing my mind. It’s normal”.*

### Category II. Trial level results

This category of results comprised two main themes: data collection methods and measures, and fidelity.

### Theme 3. Feasibility and acceptability of data collection methods and measures

#### Subtheme 3.1. Provision of a salivary sample

Biochemical verification of smoking status is standard in smoking cessation trials to evaluate intervention effectiveness. We asked participants about their experience of providing a salivary sample. Some participants found the process acceptable.

W1–P0040: *That was grand, but it got stuck in your mouth trying to get it wet. Me mouth was lovely and wet before it went in and then all of a sudden it just dried up and I wasn’t sure whether it was wet enough or not. No, it wasn’t a problem because it has to be studied.*

However, others reported that the process of providing the salivary sample was not feasible for them.

W4–P010: *It was awful. It took me ages to get a bit [of saliva]. It [the cotton swab] was very big for my mouth.*

#### Subtheme 3.2. Literacy levels

Literacy levels among participants were explored both in relation to the WCQ2 participant intervention booklet, a standard part of the programme, and paperwork associated with the trial.

W3-P0013: *The only thing that I would get you to look into is that with the writing. Too much papers, too much writing in. And I think like that for people that want to give up the cigarettes but can’t write and you might get some that can’t read and it’s embarrassing for them and that would turn them off then in going to the sessions. That’s the main thing.*

W1–P0040: *I can’t spell for diamonds, so I found it difficult if I was to write in it. One question you could put at the start [is to ask] if you have a problem filling out the forms or if you need help to complete or break down the [writing], we have no problem doing that.*

The CFs were very experienced in delivering community education programmes in SED communities so they were familiar and sensitive to low literacy. One CF had a background as a literacy tutor in a different role and she shared her insights:

W3–CF 2: *You can see that straight off when you go into a room because there’s the tell-tale signs, people are forgetting their glasses and forgetting their journals the second week…. they don’t realise about the journal and that can be very off-putting when a person… They can see that it’s like a workbook as well and that there’s writing to be done. And often… we always stress that this journal is yours and it’s not for us to see and what you do in it is your business…*

#### Subtheme 3.3. Use of repeated measures

As a part of the trial processes, questionnaire data were collected at baseline, and at 12-weeks and six-months post-intervention. Women reported satisfactory understanding of the necessity for multiple data collection timepoints.

W4 – P049: *Not at all, no, no with the help that I was after receiving I was more than willing…whatever I had to what I had to do to answer the questions. It’s payback.*

There was mixed acceptability relating to the process of providing a biological sample on more than one occasion, although they agreed to it, with one woman stating:

W3 – P004: *I wasn’t mad about giving the sample again because my mouth gets very dry but the girl [research assistant] explained why I needed to do it again – so I did it.*

### Theme 4. Fidelity

#### Subtheme 4.1. Tailoring sessions to trial checklist instead of intervention manual

Fidelity to the intervention manual was assessed by self-report methods through a checklist of intervention sessional components, completed after each session by the CFs [27]. Generally, CFs were accepting of this process and gave a positive reaction to the fidelity checklist:

W1–CF 1: *The evaluation is good because I was using that and then I’d turn it into my own little thing reminders you know the evaluating at the end of every group.*

However, there was a sense from the CFs that their use of the fidelity checklist went further than just a behavioural prompt for sessional content delivery and was discussed in terms of conscious efforts to change delivery of sessions.

W2–CF 2: *You kind of are watching a lot more…..because we had to chart everything and you were more inclined to try and stay on course… this time around, I made much more of an effort to stick to the plan.*

One CF noted that for her the presence of the fidelity assessment processes meant that she felt she was being ‘watched’ by the research team.

W2–CF 1: *I was following because I did feel you know our own diary, our community diary that was very much a kind of a “big brother watching” that you need to do those things.*

#### Subtheme 4.2. Acceptability of direct or indirect methods of fidelity assessment

Hypothetical scenarios were presented regarding alternative fidelity assessment methods. These included direct observational methods (e.g., having a researcher present in the room during group sessions) or indirect methods (e.g., audio recording of sessions and assessed at a later stage by the research team). There were some concerns relating to the acceptability of these proposed processes as a perceived threat to session privacy, and whether an audio recording could interfere with the dynamic of the session:

W2–CF 2: *I wouldn’t say record it because it’s personal to the women taking part. I wouldn’t mind them watching and that, but I wouldn’t fancy it being recorded.*

W2–CF 1: *Yeah, the watching wouldn’t bother me, but I think it would change the dynamic of the room if it was recorded.*

However, there were no concerns about having an independent observer changing the group dynamic from other CFs.

W3–CF 2: *I certainly wouldn’t have an issue; I can understand what the research is for… I don’t think that would have stopped anybody [from speaking].*

The issue of prior knowledge and consent relating to fidelity measurement was echoed amongst programme participants.

W2–P0006: *I wouldn’t have an issue with that as long as you were giving advance notice and there was real clarity around it.*

This pragmatic, democratic and accepting approach to fidelity was also shared amongst women in terms of indirect audio recordings. Alongside this an additional key issue around the confidentiality and safe keeping of recordings came into play.

W2–P0001: *So long as it was falling into the right hands and it was for research and was going to help people and maybe make the course better to help other people give up the cigarettes then [I’ve] no problem with it.*

This altruistic consideration recognised fidelity as a part of research evaluation of the programme itself.

## Discussion

The aim of this process evaluation was to examine the feasibility and acceptability of programme and trial related factors. Acceptable factors of the delivery of the intervention included peer-modelling, a non-judgemental environment, and CFs positive facilitation of group support. For some participants, provision of a saliva sample proved challenging. Participants valued free NRT as a facilitative mechanism for cessation, although some concerns about NRT side effects were expressed. Community pharmacists provided important guidance relating to NRT and additional support as a mechanism for cessation between programme meetings. The context of low literacy amongst some participants was a challenge for the feasibility of engagement with both intervention, and trial, related materials. Hypothetical scenarios of direct or indirect observational fidelity assessment for potential use in future DT were acceptable.

A key finding from this process evaluation was the importance of social support, with participants noting the value of peer group support. Benefits included: feeling accountable to others, strengthening and reinforcing motivation, learning successful strategies from peers, and allowing those who quit to share their experience and be a role model for others. It is encouraging then, that public health guidelines in the UK advocate for social support to be included in smoking cessation interventions [53]. Social support can foster a sense of community and promote continued smoking abstinence, with positive attitudes of others as major factors in determining programme engagement [54]. Stress is an important confounding factor that increases risk for relapse [55]. Lower social support can lead to increased smoking intensity and lower cessation and abstinence [56]. Social support can moderate stress levels after cessation, especially within SED cohorts [57].

There are different types of social support. Firstly, structural support is the presence of family/ friends/social networks within a person’s life. Secondly, functional support is the quality of those relationships. This includes emotional support (empathetic listening), and instrumental support (e.g., practical assistance/information provision). A third type of “support” (or its opposite) is the smoking behaviour of close others in the persons environment (e.g., partners, friends, and colleague’s). These three aspects of social support are closely interrelated and were reported as present and acceptable in WCQ2. These are also important factors as mechanisms for change within the theory of SEM [28] which underpins the programme.

Several community-based health behaviour change interventions have included the support of a ‘buddy’ from within participants’ existing social network, and found this to be correlated with smoking cessation [58, 59]. Although WCQ2 did not formally ask participants to select a ‘buddy’, participants reflected that some of their motivation was a desire not let down other members of the group. This type of camaraderie is typically seen in groups that have known each other a long time [60], however, it was reported as present in WCQ2 during a short 12-week period.

Previous studies have suggested that NRT use may increase if smokers are provided with free products and given the opportunity to find the NRT product most effective for them [61–63]. These strategies may reduce the social inequalities found in NRT usage [64]. Importantly the much-cited barrier of ‘NRT cost’ was removed from participants in this trial as the cost was borne by the charity responsible for developing the programme and not by the HSE. However, the different pathways to accessing free NRT between GMS and non-GMS participants in the same arm of the trial is an important contextual factor. GMS participants had to seek a prescription from their GP in advance of the pharmacist dispensing it. In some circumstances, women struggled to get appointments and approached pharmacists to fill the prescription ahead of getting it converted to a GMS prescription. This created embarrassment for these women, especially if the request was refused. This has implications for implementation of this aspect of the intervention. It highlights how this structural issue will need to be pre-empted and resolved for the programme to run more smoothly next time. It is important to note that the key solution to the problem of equal access to NRT lies in the bigger question of the two-tiered health system within Ireland, which goes beyond the scope of the current project.

Participants’ expressed concerns about the potential side effects of NRT, which are in line with previous findings [65], and may act as a barrier towards its use in the long-term, or incorrect or under-use [66, 67]. Concerns about becoming ‘addicted’ to NRT and about the health consequences associated with NRT are commonly held beliefs by many smokers and ex-smokers [68, 69]. This is despite the low risk of NRT addiction [70, 71] which is heavily outweighed by smoking risks. In three of the four waves women spoke highly of pharmacists and would often present to them between intervention sessions for additional support. Payment in this pilot trial for pharmacists’ time related to the dispensing of NRT with their professional guidance around medication usage only. Future DT research should comprehensively map and identify the interactions between participants and pharmacists, and also look at the provision of behavioural support training to participating pharmacists to standardise these interactions.

RCTs are considered the gold standard in clinical research. However, RCT participation can be challenging. Participants who are managing burdens associated with their behaviours (e.g., respiratory problems associated with smoking) could face additional burdens related to trial participation, such as trial research visits or supplementary procedures (some of which may be invasive e.g., provision of a salivary sample) and completion of trial questionnaires. Gathering repeated information over time is essential for understanding the behaviours under investigation (e.g., smoking and quitting), but also to accurately assess the intervention’s effects that are designed to change those behaviours (e.g., a programme like WCQ). Such tasks may deter trial participation. However, we found that it was both feasible and acceptable to collect repeated trial measures including questionnaire assessments and biological sampling over a 12-week period, for most participants. Retention rates were almost as good at six months as they were at the end of programme delivery (at 12-weeks: 55.4%; at 6-months: 47.7%) [30], which would suggest that participants that were retained at the end of programme delivery were happy to continue to provide trial data at 6 months. This is important for implementation of the next phase of the trial in which we will hope to recruit and retain as many participants as possible through each of the data collection timepoints.

One in six Irish adults have reading problems [72]. The relationship between literacy and participation in clinical research is poorly understood [73]. Shame and reluctance to disclose reading difficulties often accompany low literacy status [74], and may result in less literate people declining to engage in research activities that expose their poor literacy skills. Investigators may unknowingly facilitate this selection bias. The intervention materials (e.g., participant booklet, CF resource pack) were co-designed, delivered and adapted by experienced community development workers and health professionals. Programme materials were written and edited by health promotion professionals who were trained in plain English writing by NALA [47]. CFs are trained to demonstrate and deliver the core exercises in the programme without paper through interactive group work and props, (e.g., demonstrating a CO monitor). CFs received specific training in providing the first two programme sessions to build rapport with participants and show support in completing processes to take account of literacy needs.

It is now understood that GDPR has raised the bar for explicit informed consent and research transparency [75]. While responding and augmenting materials to increase accessibility is not new, in the post-GDPR era of conducting community-based trial research it does present both an ethical and a practical challenge for any trial that includes participants with low or no literacy ability. There are a number of implications arising from this area of the process evaluation that covers both the delivery of the programme, the next steps of a DT and more broadly at policy level. The programme providers (ICS) should review the programme and CF training guidance to further consider the challenges for low literacy participants and identify what additional supports are available in the community to address these. In addition, a dedicated section in the CF resource pack should be developed with suggestions to pre-empt and overcome these challenges in the programme. A future DT, through a Study Within A Trial (SWAT) could test strategies to improve processes relating to distilling informed consent and also how best to communicate complex health related information as it pertains to smoking cessation. At a policy level, the findings highlight the need to address educational inequality in public education. This structural societal issue can limit the impact of health and wellbeing programmes within particular population groups, e.g., women experiencing multiple socioeconomic disadvantages who also express a desire to stop smoking.

Fidelity to the delivery of a complex behavioural change intervention at community level is a significant challenge [76]. The strategies and techniques to monitor intervention fidelity are often omitted or poorly described [37, 77–80]. This is important because of the influence that fidelity has on trial outcomes [81], and furthermore, data on the attitudes of trial participants towards fidelity measures remains scarce. In the current study, findings indicated acceptance of fidelity measures for inclusion in the next phase of the trial.

The study had a number of strengths including the application of the UK MRC process evaluation guidance [31] within a community based smoking cessation trial.

Recently, the WHO has recognised the urgency of addressing tobacco use in women and the need for tailored interventions targeting specific groups of women [82]. This study focused on gaining the views of a population that is considered ‘hard to reach’ e.g., women from disadvantaged areas. In-depth qualitative interviews took place with both those who received the intervention and those who delivered it, eliciting views on both the programme itself and trial processes. This comprehensive approach will prove to be important should the programme require updating and/or in future research should the study go forward to a DT. The trial utilised COREQ guidelines which are the standardised reporting framework to improve transparency and clarity of reporting in qualitative research [48].

This study also had a number of limitations. Recruitment resulted in a self-selecting sample of smokers. The majority of participants that were interviewed had quit smoking and may have been unrepresentative; women who engaged, but saw themselves failing to maintain a quit attempt, may not have volunteered to be interviewed. In addition, we did not interview women at six-months follow-up which would have allowed for a greater period to reflect on their experience. A longer follow-up, however, could have introduced retrospective recall bias. The researcher, who conducted the interviews, was known to participants throughout the trial (e.g., took informed consent, conducted baseline assessments), which may have introduced some bias. There was some evidence of variation in the fidelity of the delivery of the intervention as it related to the support from the community pharmacist (e.g., Wave 4). The smoking journals that women kept were not assessed by the research team, as these were presented as confidential spaces in which women could note reflections of their smoking beliefs and behaviours. Even a sample of these journals could have elicited some interesting learnings from women as they navigated the programme and their quit attempt.

## Conclusions

Overall, both intervention and trial-related processes were deemed feasible and acceptable. Provision of free NRT was welcomed by participants, although some barriers remain for GMS-entitled women who still required a GP’s prescription to access the medication without charge. The role of the community pharmacist should be examined and mapped to understand interactions with participants between group meetings. The potential expansion of the role of the community pharmacist, should be considered. A future DT will need to address the low literacy levels of women from SED groups both in terms of intervention and trial related materials such as the PILs, consent forms and questionnaire measures.

## Supplementary Information

**Additional file 1.** 12-week follow up semi-structured interview guide, WCQ2 women participants.

**Additional file 2.** 12 week follow up semi-structured focus group guide, WCQ2 Community Facilitators.

## Data Availability

The pooled anonymised quantitative data analysed during the current study are available from the corresponding author on reasonable request. The qualitative data are not publicly available to protect the privacy and confidentiality of study participants.

## Abbreviations

SED: Socioeconomically disadvantaged population
WHO: World Health Organization
RCT: Randomised controlled trial
NRT: Nicotine replacement therapy
WCQ2: We Can Quit2
WCQ: We Can Quit
CFs: Community facilitators
DT: Definitive trial
UK MRC: UK Medical Research Council
HSE: Health Service Executive
GMS: General Medical Scheme
GP: General practitioner
