# Supplementary material for: A process evaluation of ‘We Can Quit’: a community-based smoking cessation intervention targeting women from areas of socio-disadvantage in Ireland
Source: BMC Public Health. 2022 Aug 10;22:1528. doi: 10.1186/s12889-022-13957-5 (PMC9367164; doi:10.1186/s12889-022-13957-5)
Supplement: Supplementary file 1 — Additional file 1. 12-week follow up semi-structured interview guide, WCQ2 women participants. [file 12889_2022_13957_MOESM1_ESM.docx]

**Additional file 1:** **12-week follow up semi-structured interview guide, WCQ2 women participants**

**Note to researcher** - Before focus group begins:

* Explain the nature of the interview to participant (voluntary, free to stop taking part at any time, research questions, why their perceptions/opinions are important, no right/wrong answers, what the data will be used for)

**Housekeeping:**

- Recording, transcribed word for word
- Phones on silent
- Thank participant for agreeing to take part
- Start recorder

**Note: The blue writing is the questions from the 12-Week FU Quantitative Questionnaire.**

**Qu 6. Questionnaire: What helped you most in trying to stop smoking? Please rank your 3 choices from 1 to 3 with 1 most helpful) NRT, Group Support, One-to-one support,**

**Qu1:** From your questionnaire, you completed with me (last week) I see you marked … as your number one choice, (*NRT or group support* as worked best)

Can you tell me why you think that was most helpful? ….

**Qu2:** Is there anything else that you feel worked well or enjoyed in the programme?

1. What did you think of the Group Support?
2. What did it mean to you meeting other smokers who want to quit?
3. What did it mean to you to get free NRT?
4. Can you tell me what the one to one support, such as text messages/telephone calls was like?
5. How did it feel having the social support of other women in the group?
6. The group is facilitated by two CFs what was your experience of having the group facilitated this way?
7. Do you set a quit date, ‘passport to quit’? Can you tell me more about that? How did it help you quit? (*Did it help with increasing and maintaining motivation to quit/stay quit)*
8. How suitable was the location of the programme? Was it close to your home?

**Qu3**: Is there anything about the programme you didn’t enjoy/ felt didn’t work so well for you?

- How did you find being in a group setting? *(Sometimes I felt that ‘my voice’ was lost in the group).*
- Thinking back, what did you think of the first session?
- What did you think of the programme being 12 weeks?
- ***Also see Relapse Question if participant brings it up here***

**Topics not covered in response to above, ask the following questions:**

**Qu 4.** Can you tell me your views on the following aspects of the WCQ Programme:

1. What did you think of the sessions conducted in a group format with other women?
2. How did you find the delivery of the programme by CF? (Use names)
3. Did you receive support of CF during one-to-one time (e.g., face-to-face, support in the meetings, Telephone calls, or texts)
4. What did you think of the Session content (e.g., setting a quit date, increasing your motivation to quit?)
5. Is there anything missing from the Programme that we need to include in future?
6. If we were to reduce/cut the programme down to its most essential parts, what do you think we would need to 1. Keep and 2. What could we cut?
7. Did you enjoy the flexibility to influence content of the last 6 weeks of Programme?
8. How did you find the access to free NRT, any difficulties accessing NRT at GP or pharmacy?
9. Did you feel you got support from the community pharmacist? How did that make you feel?
10. What did you think of a12-week duration of Programme and its Location?
11. Did you find there was Flexibility to talk about life in general besides smoking?

Can you tell me more about that? How did you feel about being able to discuss life in general besides smoking? *(e.g., relationships, children, home life, financial concerns, security of housing situation etc)*

1. Why do you think the course was women only what did you think of that,? What are if any the benefits or drawbacks to this approach?

Would you have considered a mixed group support session?

**(Qu 3 Questionnaire: Have you cut down since starting the programme?)**

**If participant has not quit**

**Qu 5:** So from your questionnaire you did (last week) you told me you have not quit smoking yet, is there anything that we could change in the future Programmes that could have helped you more?

| **Relapse: (Qu 4 Questionnaire: If you stopped smoking and relapsed, how long did you stop for? What was the main reason for your relapse? What helped you get back on track?)**  On your questionnaire you ticked you relapsed.  Could you tell me more about this?  How did you feel telling the CF or Group about your relapse?  *(I was embarrassed in front of the others that I haven’t quit yet)* |
| --- |

**Research trial processes questions:**

**Qu. 17, 18, 19, 20, 21 Questionnaire**

**(How satisfied were you with the following: Registration process / Consent process / Support received from facilitators/ Length of the programme/ Follow-up process: Ans: Very satisfied, Satisfied, Neither satisfied nor dissatisfied, Dissatisfied, Very dissatisfied).**

***(If participant has marked dissatisfied or very dissatisfied explore further)***

**Qu 6:** I can see from you questionnaire you ticked (dissatisfied/very dissatisfied) what was it about the ‘process’ that made you mark it this way?

**Qu 7:** How was your experience with the collection of data by the team?

For example, the online registration form, having to meet for signing consent and carrying out questionnaires at the beginning, and again after the 12 weeks.

- What did you think about the paper work?
- And thinking back to the start when you signed up, what did you think of the participant information sheet? *(prompt detailed* )

**Qu 8:** Was there a long time from your initial interest until the programme began? Was this an issue for you? *loss of motivation?*

**Qu 9:** What did you think about having your breathing tested/carbon monoxide (CO) reading done during appointments?

- Was it helpful to know your CO score?
- Did you understand why we were interested in your CO score?
- Were you surprised at your score the first time it was taken?
- Did you find the process of providing your CO level comfortable? (*Or a bit intrusive?)*

**(Qu 22 Questionnaire: How comfortable were you with your saliva sample being taken?)**

**Qu 10:** What did you think about having your saliva sample taken?

- How did you find the experience?

**Qu 11:** Do you have any fears or concerns about stopping the programme?

- Will you keeping in touch with other people in the group?
- Continuing to look after your health etc.

**Final question:**

**Qu 16:** Is there anything else that you would like to say that you haven’t had a chance to tell us about? **(important to ask from the participants point of view, to wrap up the interview)**

**Note to researcher - Wrap up**

Thank participant for their time and willingness to participate, close focus group

Turn off recorder
