# Supplementary material for: A process evaluation of ‘We Can Quit’: a community-based smoking cessation intervention targeting women from areas of socio-disadvantage in Ireland
Source: BMC Public Health. 2022 Aug 10;22:1528. doi: 10.1186/s12889-022-13957-5 (PMC9367164; doi:10.1186/s12889-022-13957-5)
Supplement: Supplementary file 2 — Additional file 2. 12 week follow up semi-structured focus group guide, WCQ2 Community Facilitators. [file 12889_2022_13957_MOESM2_ESM.docx]

**Additional file 2:** **12 week follow up semi-structured focus group guide, WCQ2 Community Facilitators**

**Note to researcher - Before focus group begins:**

- Explain the nature of the focus group to participants (voluntary, free to stop taking part at any time, research questions, why their perceptions/opinions are important, what the data will be used for)
- Housekeeping:
  - Recording, transcribed word for word
  - Phones on silent
  - Only one voice at a time, everyone opportunity to speak
  - Provide Participant Information Sheet and Consent form (e.g., confidentiality, anonymity – changing of names)
- Ask participants to sign the consent form, which will be countersigned by the researcher, retain consent forms
- Thank participants for agreeing to take part
- Start recorder

**Introduction:**

*For the purposes of the tape can you say your name and tell me something about you like – what’s your favourite animal. I will start. My name is Emma and my favourite animal is…….*

**Programme specific questions:**

**1. Can you tell me about your experience of being a Community Facilitator on the WCQ Programme?**

**Note to researcher: For topics not covered in response to above, ask the following questions:**

- Why did you get involved in the first place?
- Can you tell me about the training provided by ICS?
- How you see your role as a CF,
- Can you tell me what it is like having a CF ‘partner’ to work with in delivering the sessions?
- What is your opinion of the Flexibility in session content in last 6 sessions of Programme?
- What did you think of the Location/times of sessions?
- How did you find covering core session content ?
- Did you find Challenges of encouraging and motivating women who are struggling to quit/stay quit?

*2.* **What are 3 things about the WCQ Programme that you think works the best to help women stop smoking***?*

(Prompts if needed):

- meeting other smokers who want to quit,
- the social support of other women in the group,
- having the sessions close to their home,
- the ‘peer-to’peer’ Community Facilitator role,
- free NRT, setting a quit date, ‘passport to quit’,
- help with increasing and maintaining motivation to quit/stay quit, feedback on CO monitoring)

**3. What are 3 things about the WCQ Programme that you think doesn’t work as well/needs more development?**

(Prompts if needed):

- covering all of the core session content in the time allowed,
- making sure that no one individual dominates the group
- confidentiality of group discussions,
- 12 week duration too short/too long,
- maintaining motivation of women who are struggling to quit
- how did you find facilitating group conversations that are outside the topic of smoking e.g., home life, relationships, children, security of housing situation, financial pressures etc)( CF- skills/confidence)

**4. Is there anything missing from the WCQ Programme that we need to include in future?**

**5. If we were to reduce/cut the programme down to its most essential parts, what do you think we would need to 1. Keep and 2. What could we cut?**

**Research trial process questions:**

This particular WCQ Programme was different form ones that you have been involved in previously, as the Research Team from Trinity are evaluating the Programme. We would like to know about your experiences of being a part of the research.

**6. Did the fact that the Programme was going to be researched/evaluated make you ‘think twice’ about being a CF this time around?**

**7. The recruitment process was different to recruiting to a standard WCQ Programme as the women were being recruited into a trial. Do you think that this made a difference to the recruitment process? And if so how?**

(Prompts if needed):

- there was 50/50 chance to receive WCQ Programme or HSE Programme
- some women found that being part of a research trial off-putting,
- some women found that fact it was ‘Trinity’ off putting

**8. Due to the trial design areas were randomised to receive the WCQ Programme of the HSE smoking cessation Programme. This meant that the randomisation of areas happened quite close to the delivery of the Programme starting. What impact, if any, did this have on you?**

(Prompts if needed):

- Not knowing which area would get the WCQ Programme,
- State of ‘readiness’ to deliver the Programme

**9. Did you do anything differently in this WCQ Programme to try and encourage the women to continue to attend the Programme?**

(Prompts if needed):

- extra persuasion used, additional phone calls, texts etc

**10. We asked you to engage with additional paperwork (e.g., changes to the attendance record, checklist of delivery of session content), how did you find this process?**

**(Prompts if** **needed):**

- burdensome, unclear who to submit paperwork to
- didn’t understand the need/point of some of the information being asked for

**11. Do you feel that you got enough support from the Research Team throughout trial phase?**

**12. Is there anything that we could do differently in a future trial to make it easier for CF and the women taking part?**

**13. For If we were to sit in or record a session for the research team to evaluate the program?**

**Final question:**

**13. Is there anything else that you would like to say that you haven’t had a chance to tell us about?**

**Note to researcher - Wrap up**

Thank participants for their time and willingness to participate, close focus group

Turn off recorder
